# Effects of Virtual Reality Training and Whole-Body Vibration Training on Fall-Related Functional Outcomes in Older Adults: A Systematic Review and Meta-Analysis

**DOI:** 10.3390/healthcare14152409

**Published:** 2026-08-05

**Authors:** Ziyin Cao, Rudan Xi, Haiqing Zeng, Ying Tao, Yanfeng Chen, Xuewen Tian, Xiuxiu Wang

**Affiliations:** School of Sports and Health, Shandong Sports University, Jinan 250102, China; caoziyin@stu.sdpei.edu.cn (Z.C.); xirudan@stu.sdpei.edu.cn (R.X.); zenghaiqing@stu.sdpei.edu.cn (H.Z.); taoying@stu.sdpei.edu.cn (Y.T.); chenyanfeng@stu.sdpei.edu.cn (Y.C.)

**Keywords:** VR training, WBV training, older adults, falls, balance

## Abstract

**Highlights:**

**What are the main findings?**

- Both VR and WBV training improved TUG performance, indicating overlapping benefits for functional mobility and dynamic balance.
- VR additionally improved handgrip strength, Tinetti scores, and gait speed, whereas WBV improved five-times sit-to-stand performance.

**What are the implications of the main findings?**

- VR may be more suitable for improving balance, gait performance, and grip strength in older adults at risk of falls.
- WBV may be more effective for enhancing lower-limb functional strength, supporting its use as a targeted adjunct in fall-prevention rehabilitation.

**Abstract:**

Objective: To evaluate the effects of virtual reality (VR) and whole-body vibration (WBV) training on muscle strength and balance-related functional outcomes in older adults. Methods: PubMed, Web of Science, the Cochrane Library, CNKI, and Embase were searched from inception to March 2026. Randomised controlled trials assessing VR or WBV training in samples with a mean participant age of ≥60 years were included. Outcomes were the Timed Up and Go (TUG) test, handgrip strength, Tinetti score, five-times sit-to-stand test, and gait speed. Pooled mean differences (MDs) with 95% confidence intervals (CIs) were calculated. Subgroup analyses and Bucher-adjusted indirect comparisons were used to explore potential relative effects. Results: Thirty-two trials involving 1359 participants were included. VR training reduced TUG completion time (MD = −1.13, 95% CI −1.70 to −0.55, *p* = 0.0001) and improved handgrip strength (MD = 3.49, 95% CI 0.46 to 6.52, *p* = 0.02), Tinetti scores (MD = 0.84, 95% CI 0.46 to 1.21, *p* < 0.0001), and gait speed (MD = 0.16, 95% CI 0.07 to 0.24, *p* = 0.0003). WBV training reduced TUG completion time (MD = −3.92, 95% CI: −6.36 to −1.48, *p* = 0.002) and five-times sit-to-stand completion time (MD = −1.20, 95% CI −2.03 to −0.38, *p* = 0.004). Conclusions: VR and WBV training may improve functional mobility and dynamic balance in older adults, although their effects may vary across functional domains. Because direct head-to-head trials are lacking, comparative findings should be interpreted as exploratory evidence.

## 1. Introduction

Falls are common adverse events among older adults and represent a major public health concern. Epidemiological data showed that approximately 21% of individuals aged 60 years and older in China experienced falls in 2018 [1]. According to the Global Burden of Disease Study 2017, falls resulted in more than 75,000 deaths among adults aged 70 years and older worldwide in 2017. With advancing age, older adults commonly experience progressive declines in neuromuscular control, balance regulation, and proprioceptive function, all of which may contribute to an increased risk of falling [2,3,4]. Beyond fractures and soft tissue injuries, falls may also lead to functional decline, restricted mobility, loss of independence, and reduced quality of life [5]. The development of effective, sustainable, and clinically feasible strategies for fall prevention has become an important priority in rehabilitation medicine [6,7].

Strength and balance training have been widely used to improve physical function in older adults [8,9]. However, conventional training approaches still have several limitations in clinical and community-based rehabilitation settings. For example, some programs are relatively monotonous and lack real-time feedback or contextualized stimuli, which may reduce older adults’ motivation to engage in sustained training. In addition, these interventions often rely heavily on therapist guidance and supervision, with limited individualized adjustment, thereby restricting their long-term implementation in home-based and community settings [10]. These factors may reduce training adherence and attenuate long-term intervention effects. Therefore, technology-assisted rehabilitation approaches with greater interactivity, acceptability, and feasibility have received increasing attention.

Virtual reality and whole-body vibration are two representative technology-assisted interventions that have been increasingly used to support functional rehabilitation in older adults. Although both approaches aim to improve balance function and reduce fall-related functional limitations, they appear to operate through different mechanisms. VR training provides visual feedback, task-oriented practice, simulated environments, and gamified interaction, thereby facilitating sensorimotor integration, postural control, and dynamic balance regulation. In contrast, WBV training delivers mechanical vibration stimuli that activate muscle spindles and proprioceptive pathways, inducing neuromuscular responses, promoting motor unit recruitment, and enhancing lower-limb muscle activation. These mechanism-specific effects suggest that VR and WBV may influence fall-related functional outcomes through different pathways, thereby warranting further comparative evaluation.

Previous meta-analyses have examined the effects of VR [11,12,13,14] and WBV [15,16] training separately on balance function, muscle strength, and fall-related outcomes in older adults. However, most evidence has focused on comparisons with control conditions, and direct comparisons between VR and WBV remain limited. Therefore, their comparative effects are still unclear. This systematic review and meta-analysis aimed to evaluate the effects of VR and WBV training on muscle strength, balance function, and fall-related functional outcomes in older adults. In the absence of direct head-to-head trials, subgroup analyses and the Bucher-adjusted indirect comparison method were used to explore the potential relative effects of the two interventions. Given the exploratory nature of these indirect comparisons, the findings should be interpreted with caution and validated in future high-quality head-to-head randomized controlled trials.

## 2. Materials and Methods

### 2.1. Registration Information

This review was conducted and reported in accordance with the Preferred Reporting Items for Systematic Reviews and Meta-Analyses (PRISMA 2020) guidelines [17]. The study protocol was prospectively registered in PROSPERO under the registration number CRD420251250139.

### 2.2. Search Strategy

The literature search was independently conducted by two researchers in five electronic databases: PubMed, Web of Science, the Cochrane Library, CNKI and Embase. Relevant studies were searched from database inception to March 2026. The search terms included older adults, virtual reality, whole-body vibration, falls, balance, muscle strength, gait, functional mobility, and randomized controlled trials. In addition, the reference lists of relevant reviews and meta-analyses were manually screened to minimize the risk of missing eligible studies and to improve the comprehensiveness of the search. The detailed search strategy is presented in Table 1.

### 2.3. Inclusion and Exclusion Criteria

This meta-analysis was conducted in accordance with the PICOS framework. The inclusion criteria were defined as follows. (1) Population: Adults with a mean age of ≥60 years, regardless of sex or nationality, without absolute contraindications or cognitive impairment that would preclude participation in VR or WBV training. (2) Intervention: VR or WBV training as the sole experimental intervention. (3) Comparators: sham vibration, no intervention, or conventional strength and/or balance training, with an intervention duration of at least 3 weeks. (4) Outcomes: The primary outcome was the Timed Up and Go (TUG) test, which was used to assess functional mobility and fall risk; secondary outcomes included grip strength, the five-times sit-to-stand test, Tinetti score, and gait speed. (5) Study design: Randomized controlled trials published as original articles in Chinese or English with extractable data.

The exclusion criteria were as follows: studies involving participants with neurological disorders, such as stroke or Parkinson’s disease; studies with non-randomized controlled trial designs, including reviews, case reports, and cross-sectional studies; studies in which the experimental group received combined interventions involving multiple training modalities; and studies with missing or non-extractable data on muscle strength or balance-related outcomes.

### 2.4. Literature Screening and Data Extraction

All retrieved records were imported into EndNote X9 for duplicate removal. Two researchers independently screened the records according to the predefined inclusion and exclusion criteria. Any discrepancies were resolved through discussion, and a third reviewer was consulted when consensus could not be reached.

For the eligible studies, two researchers independently extracted the following information: first author, year of publication, country, sample size, intervention type, intervention duration, intervention frequency, comparator, and main findings. The outcome measures extracted for this review included the Timed Up and Go (TUG) test, grip strength, the five-times sit-to-stand test, Tinetti score, and gait speed. After data extraction, all extracted information was cross-checked by the two researchers to ensure accuracy and consistency.

### 2.5. Assessment of Risk of Bias and Quality of Included Studies

The risk of bias in the 32 included randomized controlled trials was assessed using the revised Cochrane risk-of-bias tool for randomized trials (RoB 2). The assessment covered five domains: bias arising from the randomization process, bias due to deviations from intended interventions, bias due to missing outcome data, bias in measurement of the outcome, and bias in selection of the reported result. Each domain was judged as having a low risk of bias, some concerns, or a high risk of bias.

The certainty of evidence for each main outcome was assessed using the Grading of Recommendations Assessment, Development and Evaluation (GRADE) approach. The certainty of evidence was rated as high, moderate, low, or very low based on five domains: risk of bias, inconsistency, indirectness, imprecision, and publication bias. Downgrading decisions were made according to the overall body of evidence for each outcome and were resolved by consensus among the reviewers.

Publication bias was assessed using funnel plots and Egger’s regression test when 10 or more studies were available for a given outcome within the same intervention subgroup.

**Figure 1:**
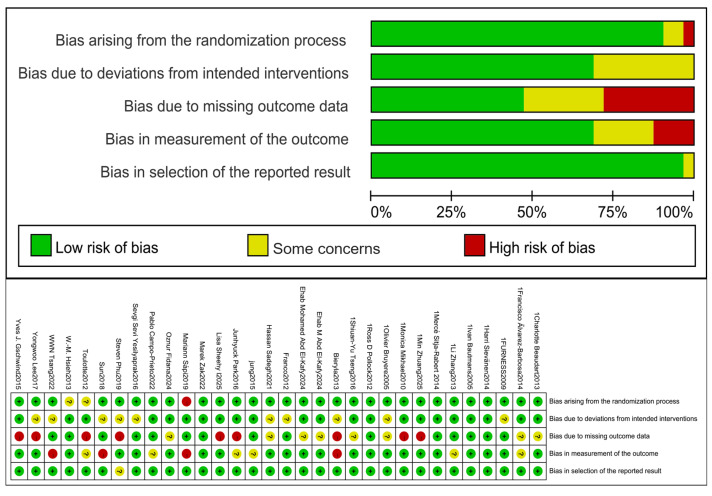
Risk-of-bias assessment of the included studies (overall risk-of-bias judgments across domains and risk-of-bias judgments for each study across domains) [18,19,20,21,22,23,24,25,26,27,28,29,30,31,32,33,34,35,36,37,38,39,40,41,42,43,44,45,46,47,48,49].

The results of the risk-of-bias assessment are presented in Figure 1. The GRADE evidence profiles are provided in the Supplementary Material S1.

### 2.6. Sensitivity Analysis

For outcomes with substantial heterogeneity, leave-one-out sensitivity analyses were conducted by sequentially removing one study at a time and recalculating the pooled effect estimates and I^2^ values. The robustness of the results was assessed according to changes in effect direction, statistical significance, and heterogeneity after study exclusion.

### 2.7. Statistical Methods

Meta-analyses were performed using Review Manager (RevMan) version 5.4 and Stata 19. All outcomes included in the quantitative synthesis were continuous outcomes. Mean differences (MD) with 95% confidence intervals (CI) were calculated when outcomes were measured using the same instruments and reported in the same units. Standardized mean differences (SMD) with 95% CI were used when different instruments or scales were applied to assess the same functional domain. The direction of effect estimates was predefined to ensure consistent interpretation across outcomes. For the TUG test and the five-times sit-to-stand test, values less than 0 favored the experimental intervention, whereas for grip strength, Tinetti score, and gait speed, values greater than 0 favored the experimental intervention.

Between-study heterogeneity was assessed using Cochran’s Q test and the I^2^ statistic. A random-effects model was applied when substantial heterogeneity was present, defined as *p* < 0.10 for Cochran’s Q test or I^2^ > 50%; otherwise, a fixed-effect model was used. Prespecified subgroup analyses were conducted according to intervention modality, namely VR training and WBV training.

Because no head-to-head randomized controlled trials directly comparing VR training with WBV training were identified, the relative effects of the two interventions were explored using the Bucher-adjusted indirect comparison method. Indirect effect estimates and corresponding 95% CI were calculated using the pooled effect estimates and standard errors from the VR-versus-control and WBV-versus-control comparisons. Given the variability in control conditions across the included studies, including no intervention, conventional training, and sham intervention, the assumptions required for valid indirect comparison may not have been fully met. Therefore, the Bucher-adjusted indirect comparisons were interpreted as exploratory and hypothesis-generating rather than as confirmatory evidence of comparative superiority. All statistical tests were two-sided, and a *p* value < 0.05 was considered statistically significant.

$$Effect_{indirect}=Effect_{VR-control}-Effect_{WBV-control}$$

$$SE_{indirect}=\sqrt{SE_{VR-control}^{2}+SE_{WBV-control}^{2}}$$

$$95\%CI=Effect_{indirect}\pm 1.96\times SE_{indirect}$$

## 3. Results

### 3.1. Literature Search Results and Flowchart

**Figure 2:**
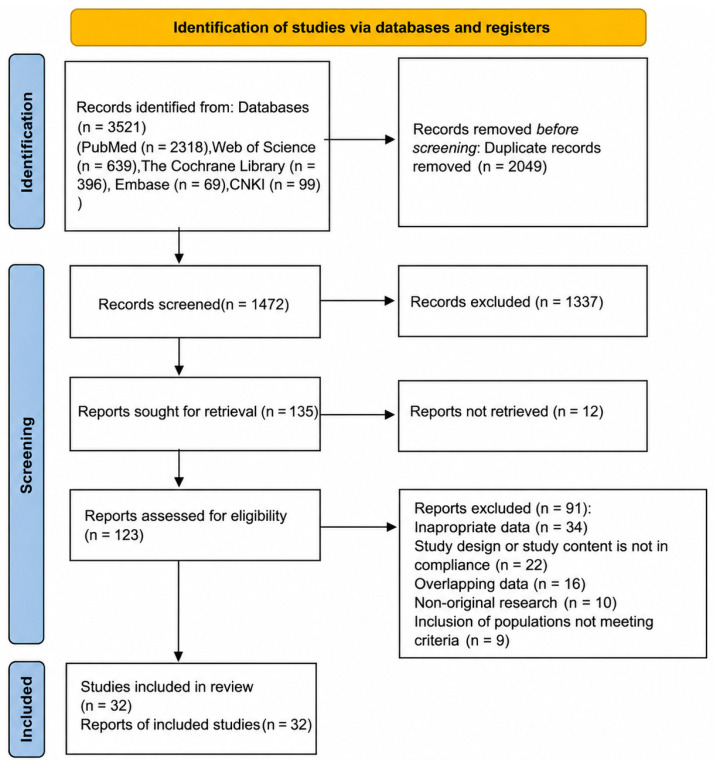
PRISMA flow diagram of study selection. Abbreviation: PRISMA, Preferred Reporting Items for Systematic Reviews and Meta-Analyses.

A total of 3521 articles were initially screened. After importing them into “EndNote X9” to remove duplicates, 1472 articles remained. Further screening based on titles and abstracts excluded 1337 articles unrelated to the study topic, leaving 135 articles. Full-text screening identified 32 articles meeting the requirements for meta-analysis. The screening process is shown in Figure 2.

### 3.2. Characteristics of Included Studies

A total of 32 studies were included in this review, including 12 studies investigating WBV training (Table 2) and 20 studies investigating VR training (Table 3). The VR training studies included 869 participants in total, comprising 445 participants in the VR groups and 424 participants in the control groups. The WBV training studies included 490 participants in total, comprising 241 participants in the WBV groups and 249 participants in the control groups. Across the included randomized controlled trials, variations were observed in VR equipment, WBV equipment, intervention frequency, and intervention duration.

### 3.3. Sensitivity Analyses

For the effect of VR training on the five-times sit-to-stand test, heterogeneity decreased from 67% to 0% after excluding Campo-Prieto et al.; however, the pooled effect reached only borderline statistical significance (MD = −1.29, 95% CI: −2.58 to 0.00, *p* = 0.05). Similarly, for the effect of VR training on gait speed, heterogeneity decreased to 0% after excluding Sadeghi et al. (I^2^ decreased from 69% to 0%), and the pooled effect remained statistically significant (MD = 0.11, 95% CI: 0.06 to 0.17, *p* < 0.0001). These findings suggest that the studies by Campo-Prieto et al., and Sadeghi et al. may have contributed substantially to the heterogeneity observed in the VR training analyses for the five-times sit-to-stand test, and gait speed, respectively. After excluding these potential sources of heterogeneity, the direction of the pooled effects remained generally consistent, and the results were statistically significant or borderline significant. This indicates that the findings were relatively stable. Nevertheless, given the limited number of studies included for these outcomes and the borderline significance observed for the five-times sit-to-stand test, the sensitivity analysis findings should be interpreted with caution. The corresponding forest plots after study exclusion are provided in Supplementary Figures S3 and S4.

### 3.4. Meta-Analysis Results

#### 3.4.1. TUG Test

**Figure 3:**
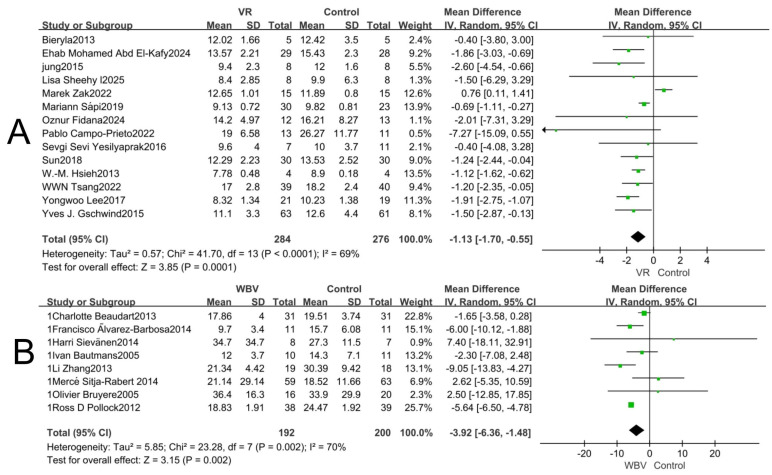
Forest plots of meta-analyses: (**A**) VR TUG test; (**B**) WBV TUG test; CI, confidence interval [18,19,20,21,22,23,24,25,26,27,28,29,30,31,32,33,34,35,36,37,38,39].

A total of 22 studies reported TUG outcomes, including 14 studies involving VR training [18,19,20,21,22,23,24,25,26,27,28,29,30,31] and 8 studies involving WBV training [32,33,34,35,36,37,38,39], with a total of 952 participants. The corresponding forest plots are shown in Figure 3. Among the 14 VR studies, the VR groups included 284 participants and the control groups included 276 participants. Substantial between-study heterogeneity was observed (χ^2^ = 41.70, df = 13, *p* < 0.001, I^2^ = 69%); therefore, a random-effects model was applied. The pooled results showed that VR training significantly reduced TUG completion time compared with control conditions (MD = −1.13, 95% CI: −1.70 to −0.55, *p* = 0.0001), suggesting improvements in functional mobility and dynamic balance performance (Figure 3A).

Among the 8 WBV studies, the WBV groups included 192 participants and the control groups included 200 participants. Substantial heterogeneity was observed across studies (χ^2^ = 23.28, df = 7, *p* = 0.002, I^2^ = 70%); therefore, a random-effects model was applied (Tau^2^ = 5.85). The pooled results showed that WBV training also significantly reduced TUG completion time compared with control conditions (MD = −3.92, 95% CI: −6.36 to −1.48, *p* = 0.002) (Figure 3B).

Further exploratory analysis using the Bucher-adjusted indirect comparison suggested a larger reduction in TUG completion time with WBV training than with VR training (indirect MD = 2.79, 95% CI: 0.28 to 5.30, *p* = 0.029). Nevertheless, because this comparison was not based on direct head-to-head randomized controlled trials and the control conditions varied across the included studies, this finding should be interpreted as exploratory rather than as definitive evidence of the superiority of WBV over VR.

To further explore the source of heterogeneity in the WBV subgroup, an additional subgroup analysis was conducted according to intervention duration. The results suggested that intervention duration may partly explain the heterogeneity in the TUG outcome. When the intervention duration was ≤8 weeks, WBV training significantly reduced TUG completion time (Z = 4.10, *p* < 0.0001), although moderate heterogeneity was observed among the studies (I^2^ = 45%). In contrast, studies with an intervention duration >8 weeks did not show a statistically significant reduction in TUG completion time (Z = 1.63, *p* = 0.10), and no apparent heterogeneity was observed among these studies (I^2^ = 0%). The test for subgroup differences was statistically significant (χ^2^ = 4.61, *p* = 0.03), suggesting that intervention duration may be one source of heterogeneity. However, because the >8-week subgroup included only two studies, this finding should be interpreted with caution and should not be considered evidence that longer intervention durations are ineffective. The corresponding forest plot is provided in the Supplementary Material S5.

In the VR TUG analysis, a leave-one-out sensitivity analysis showed that heterogeneity decreased substantially after excluding Marek Zak et al. (I2 decreased from 69% to 16%). However, the exclusion of this study did not materially alter the magnitude, direction, or statistical significance of the pooled effect, indicating that the overall finding was robust. The corresponding forest plot is provided in Supplementary Material S7. The results of Egger’s test for VR TUG outcomes are presented in Table 4.

#### 3.4.2. Grip Strength

**Figure 4:**
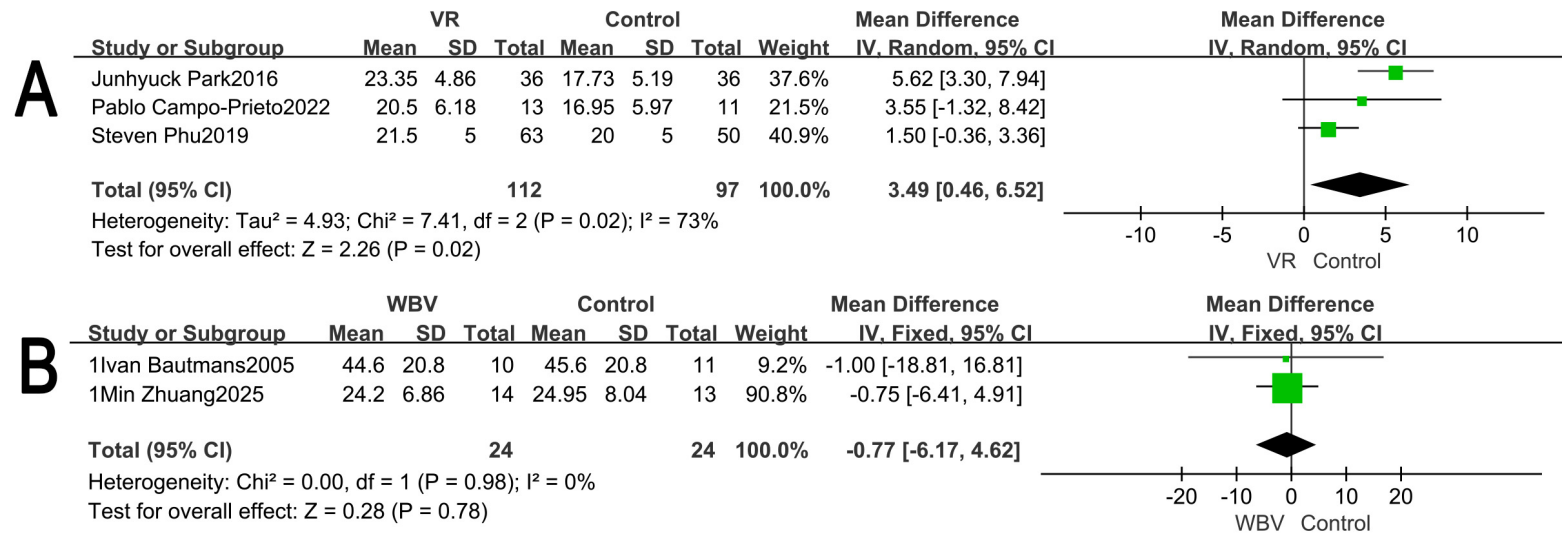
Forest plots of meta-analyses: (**A**) VR grip strength test; (**B**) WBV grip strength test. CI, confidence interval [25,35,40,41,42].

A total of five studies reported grip strength outcomes, including three VR training studies [25,40,41] and two WBV training studies [35,42], involving 257 participants. Grip strength was assessed using a handheld dynamometer for the dominant hand, and the average value of three repeated measurements was recorded. The corresponding forest plots are shown in Figure 4.

In the three VR studies, the VR groups included 112 participants and the control groups included 97 participants. Substantial heterogeneity was observed among the studies (χ^2^ = 7.41, df = 2, *p* = 0.02, I^2^ = 73%); therefore, a random-effects model was applied (Tau^2^ = 4.93). The pooled results showed that VR training significantly improved grip strength compared with control conditions (MD = 3.49, 95% CI: 0.46 to 6.52, Z = 2.26, *p* = 0.02) (Figure 4A).

In the two WBV studies, the WBV groups and control groups each included 24 participants. No evidence of between-study heterogeneity was observed (χ^2^ = 0.00, df = 1, *p* = 0.98, I^2^ = 0%); therefore, a fixed-effect model was used. The pooled results showed that WBV training did not significantly improve grip strength compared with control conditions (MD = −0.77, 95% CI: −6.17 to 4.62, Z = 0.28, *p* = 0.78) (Figure 4B).

The Bucher-adjusted indirect comparison showed no statistically significant difference between VR and WBV training in grip strength improvement (indirect MD = 4.25, 95% CI: −1.94 to 10.44, *p* = 0.179). Therefore, although VR training showed a significant improvement compared with control conditions whereas WBV training did not, the current indirect evidence is insufficient to conclude that VR training is superior to WBV training for improving grip strength. This finding should be interpreted with caution because only two WBV studies were included, with a limited sample size of 24 participants per group. Owing to the lack of direct head-to-head comparisons, the findings from the Bucher-adjusted indirect comparisons should be regarded as exploratory evidence.

However, for the effect of VR training on grip strength, heterogeneity decreased substantially after excluding Park et al. (I^2^ decreased from 73% to 0%). The pooled effect remained borderline statistically significant the effect size was attenuated, and the confidence interval approached the null (MD = 1.76, 95% CI: 0.03 to 3.49, *p* = 0.05), indicating that the overall estimate was sensitive to the inclusion of this study and should therefore be interpreted with caution. The corresponding forest plot is provided in Supplementary Material S2.

#### 3.4.3. Five-Times Sit-to-Stand Test

**Figure 5:**
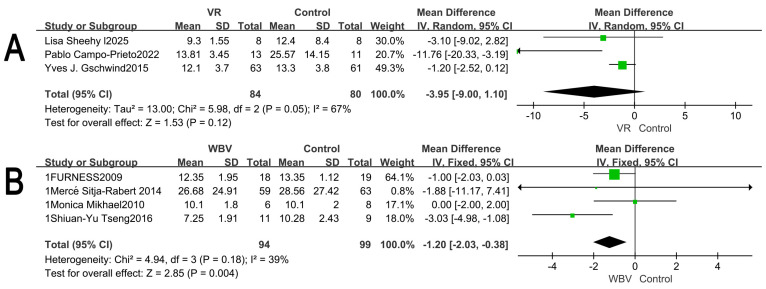
Forest plots of meta-analyses: (**A**) Five-Times Sit-to-Stand Test in the VR group; (**B**) Five-Times Sit-to-Stand Test in the WBV group; CI, confidence interval [21,25,31,37,43,44,45].

A total of seven studies reported outcomes for the five-times sit-to-stand test, including three VR training studies [21,25,31] and four WBV training studies [37,43,44,45], involving 357 participants. The corresponding forest plots are shown in Figure 5.

In the three VR studies, the VR groups included 84 participants and the control groups included 80 participants. Substantial between-study heterogeneity was observed (χ^2^ = 5.98, df = 2, *p* = 0.05, I^2^ = 67%); therefore, a random-effects model was applied (Tau^2^ = 13.00). The pooled results showed that VR training did not significantly reduce five-times sit-to-stand completion time compared with control conditions (MD = −3.95, 95% CI: −9.00 to 1.10, Z = 1.53, *p* = 0.12) (Figure 5A).

In the four WBV studies, the WBV groups included 94 participants, and the control groups included 99 participants. Low-to-moderate between-study heterogeneity was observed (χ^2^ = 4.94, df = 3, *p* = 0.18, I^2^ = 39%); therefore, a fixed-effect model was used. The pooled results indicated that WBV training significantly reduced five-times sit-to-stand completion time compared with control conditions (MD = −1.20, 95% CI: −2.03 to −0.38, Z = 2.85, *p* = 0.004) (Figure 5B).

The Bucher-adjusted indirect comparison showed no statistically significant difference between VR and WBV training for the five-times sit-to-stand test (indirect MD = −2.75, 95% CI: −7.87 to 2.37, *p* = 0.29). Therefore, although WBV training showed a significant improvement compared with control conditions and VR training did not, the current indirect evidence is insufficient to conclude that either intervention is superior to the other for improving five-times sit-to-stand performance. In the absence of direct comparative trials, the Bucher-adjusted results provide exploratory evidence only.

#### 3.4.4. Tinetti Score

**Figure 6:**
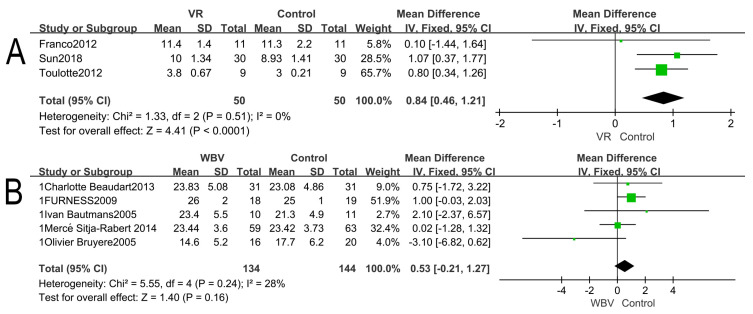
Forest plots of meta-analyses: (**A**) VR Tinetti score; (**B**) WBV Tinetti score. CI, confidence interval [27,32,35,37,38,43,46,47].

A total of eight studies reported Tinetti score outcomes, including three VR training studies [27,46,47] and five WBV training studies [32,35,37,38,43], involving 378 participants. The corresponding forest plots are shown in Figure 6.

In the three VR studies, the VR and control groups each included 50 participants. No between-study heterogeneity was observed (χ^2^ = 1.33, df = 2, *p* = 0.51, I^2^ = 0%); therefore, a fixed-effect model was used. The pooled results showed that VR training significantly improved Tinetti scores compared with control conditions (MD = 0.84, 95% CI: 0.46 to 1.21, Z = 4.41, *p* < 0.0001) (Figure 6A).

In the five WBV studies, the WBV groups included 134 participants and the control groups included 144 participants. Low between-study heterogeneity was observed (χ^2^ = 5.55, df = 4, *p* = 0.24, I^2^ = 28%); therefore, a fixed-effect model was used. The pooled results showed that WBV training did not significantly improve Tinetti scores compared with control conditions (MD = 0.53, 95% CI: −0.21 to 1.27, Z = 1.40, *p* = 0.16) (Figure 6B).

The Bucher-adjusted indirect comparison showed no statistically significant difference between VR and WBV training in improving Tinetti scores (indirect MD = 0.31, 95% CI: −0.52 to 1.14, *p* = 0.46). Therefore, although VR training showed a significant improvement compared with control conditions, WBV training did not, and the current indirect evidence is insufficient to conclude that VR training is superior to WBV training for improving Tinetti score outcomes. Without direct head-to-head evidence, the Bucher-adjusted results should be regarded as exploratory.

#### 3.4.5. Gait Speed

**Figure 7:**
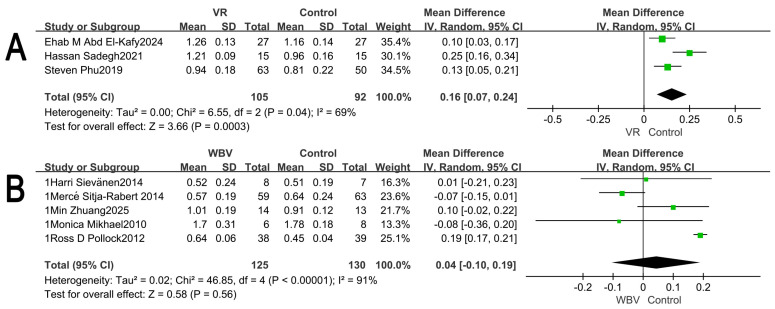
Forest plots of meta-analyses: (**A**) VR Gait Speed; (**B**) WBV Gait Speed. CI, confidence interval [34,37,39,41,42,44,48,49].

A total of eight studies reported gait speed outcomes, including three VR training studies [41,48,49] and five WBV training studies [34,37,39,42,44], involving 452 participants. The corresponding forest plots are shown in Figure 7.

In the three VR studies, the VR groups included 105 participants and the control groups included 92 participants. Substantial between-study heterogeneity was observed (χ^2^ = 6.55, df = 2, *p* = 0.04, I^2^ = 69%); therefore, a random-effects model was applied (Tau^2^ = 0.00). The pooled results showed that VR training significantly improved gait speed compared with control conditions (MD = 0.16, 95% CI: 0.07 to 0.24, Z = 3.66, *p* = 0.0003) (Figure 7A).

In the five WBV studies, the WBV groups included 125 participants and the control groups included 130 participants. Considerable between-study heterogeneity was observed (χ^2^ = 46.85, df = 4, *p* < 0.00001, I^2^ = 91%); therefore, a random-effects model was applied (Tau^2^ = 0.02). The pooled results showed that WBV training did not significantly improve gait speed compared with control conditions (MD = 0.04, 95% CI: −0.10 to 0.19, Z = 0.58, *p* = 0.56) (Figure 7B).

To explore potential sources of heterogeneity in the effect of WBV training on gait speed, a subgroup analysis was conducted according to age. In studies with a mean participant age of ≥70 years, WBV training significantly improved gait speed (MD = 0.14, 95% CI: 0.05 to 0.23, *p* = 0.004), although moderate heterogeneity remained (I^2^ = 57%). In studies with a mean participant age of <70 years, WBV training showed a borderline negative effect on gait speed (MD = −0.07, 95% CI: −0.14 to 0.00, *p* = 0.06), indicating a possible slight slowing of gait rather than an improvement. The test for subgroup differences showed a statistically significant difference between the two age subgroups (*p* = 0.0006), suggesting that age may be a potential source of heterogeneity in the effect of WBV training on gait speed. However, because this analysis was based on study-level mean age and included a limited number of studies, the findings should be interpreted as exploratory. The corresponding forest plot is provided in the Supplementary Material S6.

The Bucher-adjusted indirect comparison was further performed to explore the potential relative effects of VR and WBV training on gait speed. The results showed no statistically significant difference between the two interventions (indirect MD = 0.12, 95% CI: −0.05 to 0.29, *p* = 0.162). Therefore, despite a statistically significant improvement observed for VR training but not for WBV training when each was compared with control conditions, the current indirect evidence is insufficient to conclude that VR training is superior to WBV training for improving gait speed. As no direct head-to-head trials were available, the Bucher-adjusted conclusions remain exploratory.

### 3.5. Publication Bias

**Figure 8:**
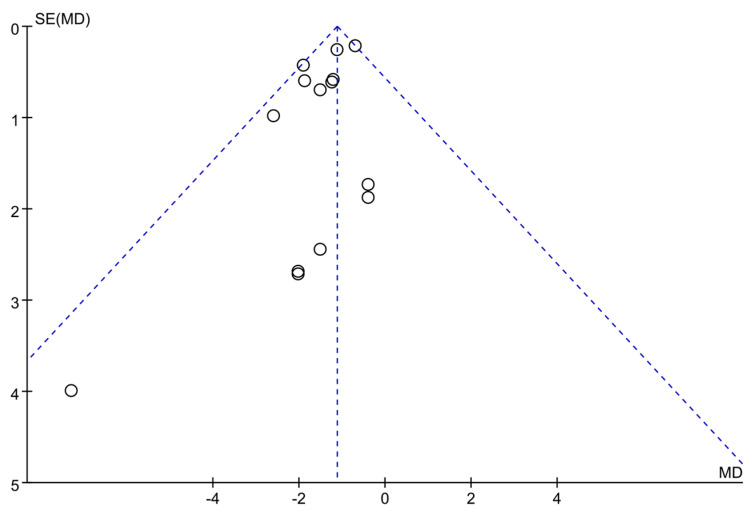
Funnel plot assessing potential publication bias for the effect of virtual reality training on Timed Up and Go performance.

Visual inspection of the funnel plot revealed slight asymmetry, suggesting potential publication bias or small-study effects; however, this finding should be interpreted with caution, as shown in Figure 8.

## 4. Discussion

This systematic review and meta-analysis examined the effects of VR and WBV training on muscle strength, balance, and functional mobility in older adults. Overall, both interventions demonstrated potential benefits, although the magnitude and pattern of their effects varied across specific functional outcomes. Importantly, both VR and WBV training significantly improved TUG performance, indicating overlapping benefits in dynamic balance and functional mobility. Beyond this shared effect, VR training was associated with improvements in handgrip strength, Tinetti scores, and gait speed, whereas WBV training was associated with improved five-times sit-to-stand performance. These outcome-specific patterns may reflect differences in the underlying mechanisms of the two interventions but should not be interpreted as indicating mutually exclusive functional effects. However, because direct head-to-head randomized controlled trials are currently lacking and most indirect comparisons did not show statistically significant differences between the two interventions, the findings of this review do not establish the definitive superiority of either intervention. Taken together, VR and WBV training may be considered technology-assisted rehabilitation approaches with partially overlapping and outcome-specific benefits, and their clinical application should be individualized according to older adults’ functional deficits, physical condition, and rehabilitation goals.

### 4.1. Muscle Strength

Regarding muscle strength outcomes, VR training significantly improved grip strength compared with control conditions, whereas WBV training did not show a significant effect. Grip strength, an important indicator of upper-limb function, has been reported to be negatively associated with fall risk in older adults, although this association has not been consistently observed [50]. This inconsistency may be partly explained by baseline characteristics, particularly age. In the VR studies reporting grip strength, participants with a mean age of <80 years had a baseline grip strength of approximately 20 kg [40,41], whereas those with a mean age of ≥80 years had a baseline value of approximately 15 kg [25], Some VR protocols require participants to wear 3D glasses and hold control handles, while gamified scenarios may enhance engagement, attentional focus, and motor learning. In contrast, WBV primarily delivers mechanical vibration stimuli through the feet and lower limbs, emphasizing lower-limb muscle spindles, proprioceptive pathways, and neuromuscular responses [51]. Thus, its effects may be concentrated on lower-limb function, postural control, and functional mobility, with limited direct stimulation of the hand and forearm muscles. Because grip strength improvement usually requires active and repetitive training of these muscles, this may partly explain why WBV did not significantly improve grip strength. Given the limited number of WBV studies reporting this outcome, the finding should be interpreted with caution.

Our analysis also showed that WBV training significantly reduced five-times sit-to-stand completion time, whereas VR training did not show a statistically significant effect. The sit-to-stand task is a fundamental transfer activity for maintaining independence in daily living, and its performance is closely related to lower-limb strength, muscular endurance, and coordinated control of the hip, knee, and ankle joints [52]. Because WBV mainly acts on the lower-limb neuromuscular system, its effects on muscle activation, proprioceptive input, and postural control may help improve sit-to-stand efficiency [53]. A shorter completion time may reflect improved lower-limb endurance and functional mobility and may reduce postural instability during the transition to standing. Owing to its passive stimulation and relatively low cognitive demand, WBV may have favorable clinical applicability, particularly for frail older adults or those with muscle weakness, impaired balance, or poor exercise adherence.

### 4.2. Balance Function

Regarding balance outcomes, the TUG test is widely used to assess dynamic balance and functional mobility because it reflects common fall-related activities, such as standing up, walking, turning, and sitting down [54]. A shorter TUG completion time indicates better mobility performance and may be associated with a lower risk of falls. In this review, both VR and WBV significantly reduced TUG completion time, consistent with previous studies [12,55]. The subgroup analysis and Bucher-adjusted indirect comparison further suggested a potential signal favouring WBV training in reducing TUG completion time. However, this finding should be interpreted as exploratory because it was not based on direct head-to-head randomized controlled trials. Given that each 1-s increase in TUG time has been associated with an approximately 9% increase in future fall risk, improving TUG performance may have important clinical relevance [56].

For Tinetti score outcomes, VR showed a significant improvement compared with control conditions, whereas WBV training did not. Nevertheless, the Bucher-adjusted indirect comparison showed no statistically significant difference between the two interventions, indicating that the current evidence is insufficient to conclude that VR training is superior to WBV for improving Tinetti scores. Overall, WBV may be more closely related to functional mobility and transfer-related dynamic balance, whereas VR may provide broader stimulation of balance control through visual feedback, task-oriented practice, and sensorimotor integration. These interpretations remain exploratory and require confirmation in future head-to-head trials.

### 4.3. Functional Ambulation

Gait speed is a comprehensive indicator of muscle strength, balance function, and functional mobility in older adults. It is closely associated with fall risk, functional independence, and long-term prognosis, and is therefore regarded as a sensitive clinical measure of health status. Previous evidence has shown that older adults with a gait speed below 1.0 m/s are at increased risk of falling [57]. In addition, individual differences in gait speed may reflect variations in balance control and are independently associated with fall risk [58]. Therefore, improving gait speed represents an important target for fall-prevention interventions in older adults.

In this review, VR significantly improved gait speed, whereas WBV did not show a statistically significant effect overall, consistent with the findings reported by Freddy et al. [55]. Notably, the subgroup of studies with a mean participant age below 70 years showed a borderline negative effect of WBV on gait speed (MD = −0.07, *p* = 0.06), suggesting a possible slight slowing of gait; however, because the confidence interval included the null value and the analysis was based on a limited number of studies using study-level mean age, this finding should be interpreted cautiously and should not be regarded as evidence that WBV adversely affects gait speed. The beneficial effect of VR may be related to its ability to simulate balance- and gait-related scenarios while providing visual, auditory, and proprioceptive feedback. Such multidimensional sensory inputs may help optimize gait rhythm, enhance dynamic balance, and improve sensorimotor integration through central nervous system plasticity, thereby contributing to increased gait speed. In contrast, WBV primarily targets lower-limb neuromuscular activation and proprioceptive responses through mechanical vibration, but may provide less task-specific gait practice than VR. This difference may partly explain why VR training significantly improved gait speed, whereas WBV training did not. However, because the Bucher-adjusted indirect comparison showed no statistically significant difference between the two interventions, the current evidence is insufficient to conclude that VR is superior to WBV for improving gait speed.

### 4.4. Limitations

Several limitations should be acknowledged in this study. First, no direct head-to-head randomized controlled trials comparing VR training with WBV training were available in the included studies. Therefore, the relative effects of the two interventions were mainly derived from subgroup analyses and Bucher-adjusted indirect comparisons, and the corresponding findings should be interpreted as exploratory evidence. Second, the included studies varied in intervention protocols, training frequency, intervention duration, equipment type, and control conditions, which may have contributed to between-study heterogeneity and affected the stability of the pooled estimates. Third, the number of studies available for several outcomes was relatively small, particularly for grip strength, the five-times sit-to-stand test, Tinetti scores, and gait speed. The limited sample sizes may have reduced statistical power and restricted the generalizability of the findings.

## 5. Conclusions

This meta-analysis suggests that both VR and WBV training may have beneficial effects on functional mobility and dynamic balance in older adults, although the magnitude and pattern of these effects may vary across specific functional domains. VR training showed potential benefits for grip strength, Tinetti scores, and gait speed, whereas WBV training showed beneficial effects on functional mobility measures, including the TUG test and the five-times sit-to-stand test.

In clinical practice, both VR and WBV training may be considered potential adjunctive strategies for fall prevention and functional rehabilitation in older adults. However, intervention selection should be individualized according to functional deficits, physical condition, training accessibility, and rehabilitation goals. Future well-designed head-to-head randomized controlled trials with adequate sample sizes and standardized control conditions are needed to further clarify the comparative effectiveness of these two interventions and identify the populations most likely to benefit from each approach.

## Figures and Tables

**Table 1:** Search terms for the literature included in this study.

Group	Subject Term
1	randomized controlled trial, controlled clinical trial, randomized, randomised, trial, groups
2	Virtual reality training; Kinect Training; Kinematic; Wii Fit training; C-Mill Virtual Reality; whole-body vibration training; Carl Zeiss VR One Plus; Jintronix; Xbox 360 Kinect; BTS NIRVANA VR; Balance Rehabilitation Unit; Motion 2.0 VR system
3	Elderly; Older Adults; Older People; Aged;

**Table 2:** Characteristics of included studies in the WBV literature.

Author (Country)	Subjects				Intervention Measures				Outcome
	Experimental/ Control	Age	Height (cm)	Weight (kg)	Intervention Exercise	Control Group	Frequency (Week)	Duration (Weeks)	
Charlotte Beaudart (Belgium)	31/31	WBV: 82.2 ± 9.02 C: 84.2 ± 6.83	WBV: 163(159–175) C: 162(158–166)	W: 68.8 ± 14.1 C: 58.9 ± 12.6	WBV	No intervention	3	12	TUG; Tinetti score
FranciscoÁlvarez-Barbosa (Spain)	11/11	WBV: 85.5 ± 6.7 C: 84.0 ± 3.0	NR	NR	WBV	No intervention	3	8	TUG
FURNESS (Australia)	18/19	T: 72 ± 8	T: 167 ± 8	T: 77 ± 12	WBV	No intervention	3	6	FTSST; Tinetti score
Harri Sievänen (Finland)	8/7	WBV: 84.4 ± 6.3 C: 83.6 ± 8.9	WBV: 160 ± 10 C: 163 ± 7	WBV: 69.1 ± 13.2 C: 75.7 ± 9.5	WBV	Pseudo-vibration	2	10	TUG; Grip strength; Gait speed
Ivan Bautmans (Belgium)	10/11	WBV: 76.6 ± 11.8 C: 78.6 ± 10.4	WBV: 163 ± 9 C: 163 ± 9	WBV: 66.7 ± 13.8 C: 63.2 ± 21.1	WBV	Pseudo-vibration	3	6	TUG; Grip strength; Tinetti score
Li Zhang (China)	19/18	WBV: 85.84 ± 3.58 C: 84.67 ± 3.68	WBV: 166.42 ± 6.20 C: 163.61 ± 4.74	WBV: 69.37 ± 3.62 C: 67.06 ± 5.87	WBV	PT	3–5	8	TUG
MercéSitja-Rabert (Spain)	59/63	>65	NR	NR	WBV	No intervention	3	6	TUG; FTSST; Tinetti score; Gait speed
Min Zhuang (China)	14/13	WBV: 73.93 ± 3.73 C: 73.154 ± 4.22	NR	WBV: 58.68 ± 9.45 C: 56.04 ± 12.61	WBV	Resistance training	3	12	Gait speed; Grip strength
Monica Mikhael (Australia)	6/8	WBV: 63.3 ± 7.6 C: 62.3 ± 8.8	WBV: 166.7 ± 9.1 C: 168.4 ± 12.0	WBV: 72.9 ± 10.4 C: 83.4 ± 12.12	WBV	Pseudo-vibration training	3	13	Gait speed; FTSST
Olivier Bruyere (Belgium)	16/20	WBV: 84.5 ± 5.9 C: 78.9 ± 6.9	NR	NR	WBV	PT	3	6	TUG; Tinetti score;
Ross D Pollock (UK)	38/39	WBV: 80 ± 8.6 C: 82 ± 8.1	NR	NR	WBV	PT	3	8	TUG; Gait speed
Shiuan-Yu Tseng (Taiwan, China)	11/9	WBV: 67.21 ± 2.29 C: 68.57 ± 2.50	WBV: 158.96 ± 6.13 C: 163.57 ± 8.70	WBV: 61.93 ± 9.47 C: 62.00 ± 10.16	WBV	No intervention	3	12	FTSST

Note: NR indicates that the information was not reported in the original study.

**Table 3:** Characteristics of studies included in the VR literature review.

Author (Country)	Subjects				Intervention Measures				Outcome
	Experimental/ Control	Age (Years)	Height (cm)	Weight (kg)	Intervention Exercise	Control Group	Frequency (Week)	Duration (Weeks)	
Bieryla (USA)	5/5	VR: 82.5 ± 1.6 C: 80.5 ± 7.8	NR	NR	Wii Fit VR	No intervention	3	3	TUG
Ehab M Abd El-Kafy (Saudi Arabia) a ^a^	27/27	VR: 66.53 ± 3.82 C: 65.43 ± 4.28	VR: 169.41 ± 5.26 C: 168.39 ± 4.28	VR: 88.74 ± 6.26 C: 89.52 ± 4.43	C-Mill	Regular Training and Running	3	4	Gait speed
Ehab Mohamed Abd El-Kafy (Saudi Arabia) b ^a^	29/28	VR: 66.53 ± 3.82 C: 65.43 ± 4.28	VR: 168.53 ± 4.72 C: 167.47 ± 3.49	VR: 86.85 ± 5.61 C: 88.34 ± 4.58	C-Mill	Regular training and running	3	6	TUG
Franco (USA)	11/11	VR: 79.8 ± 4.7 C: 76.9 ± 6.3	NR	NR	Wii FitV	No intervention	2	3	Tinetti score
Hassan Sadegh (Iran)	15/15	VR: 74.1 ± 7.0 C: 72.2 ± 7.2	VR: 166.8 ± 5.1 C: 167.2 ± 5.6	VR: 68.7 ± 8.7 C: 66.5 ± 10.5	Xbox Kinect	No intervention	3	8	Gait speed
Jung (South Korea)	8/8	VR: 74.3 ± 2.1 C: 73.6 ± 2.4	VR: 152.5 ± 4.4 C: 151.6 ± 5.4	VR: 54.9 ± 9.6 C: 55.3 ± 6.2	Wii Fit VR	No intervention	2	6	TUG
Junhyuck Park (Iran)	36/36	VR: 72.97 ± 2.98 C: 74.11 ± 2.88	VR: 155.16 ± 5.57 C: 153.64 ± 4.68	VR: 54.21 ± 5.01 C: 53.15 ± 3.97	Xbox Kinect	No intervention	3	8	Grip strength
Lisa Sheehy (Canada)	8/8	VR: 70.9 ± 4.3 C: 75.1 ± 6.0	NR	NR	Jintronix	No intervention	3	8	TUG; FTSST
Marek Zak (Poland)	15/15	VR: 76.66 ± 1.5 C: 76.66 ± 1.3	VR: 166 ± 1.82 C: 167 ± 1.85	VR: 71.2 ± 12.75 C: 71 ± 10.74	Carl Zeiss VR One Plus	Regular Training	3	3	TUG
Mariann SáPi (Hungary)	30/23	VR: 69.57 ± 4.66 C: 69.12 ± 4.19	NR	NR	Kinect	No intervention	3	6	TUG
Oznur Fidana (Turkey)	12/13	VR: 75.53 ± 8.43 C: 75.57 ± 7.88	NR	NR	Xbox 360 Kinect	No intervention	2	8	TUG
Pablo CamPo-Prieto (Spain)	13/11	VR: 85.08 ± 8.48 C: 84.82 ± 8.10	VR: 155 ± 9 C: 149 ± 8	VR: 60.32 ± 10.53 C: 61.60 ± 11.75	BOX VR	No intervention	3	10	TUG; Grip strength; FTSST
Sevgi Sevi YesilyaPrak (Turkey)	7/11	VR: 70.1 ± 4.0 C: 73.1 ± 4.5	NR	NR	BTS NIRVANA VR	Regular Training	3	6	TUG
Steven Phu (Australia)	63/50	VR: 79 ± 5 C: 79 ± 5	NR	NR	Balance Rehabilitation Unit(BRU)	No intervention	2	6	Grip strength; Gait speed
Sun (China)	30/30	VR: 67.8 ± 3.1 C: 68.4 ± 2.3	VR: 156.97 ± 8.76 C: 158.30 ± 5.48	VR: 58.74 ± 10.89 C: 54.13 ± 9.35	Motion 2.0 VR system	No intervention	3	12	TUG; Tinetti score
Toulotte (France)	9/9	VR: 70.2 ± 2.4 C: 72.1 ± 2.2	VR: 168 ± 3 C: 162 ± 9	VR: 80.5 ± 9.7 C: 76.6 ± 13.6	Nintendo Wii Fit VR system	No intervention	1	20	Tinetti score
W.-M.Hsieh (Taiwan)	4/4	VR: 60.25 ± 5.25 C: 67.25 ± 7.98	VR: 166.15 ± 13.87 C: 159.25 ± 9.92	VR: 63.75 ± 12.94 C: 68.5 ± 10.82	Kinect	No intervention	5	6	TUG
WWN Tsang (Hong Kong)	39/40	VR: 82.3 ± 3.8 C: 82 ± 4.3	VR: 154 ± 10 C: 157 ± 10	VR: 52.6 ± 4.6 C: 55.9 ± 8.5	Wii Fit	Regular Training	3	6	TUG
Yongwoo Lee (South Korea)	21/19	VR: 76.15 ± 4.55 C: 75.71 ± 4.91	VR: 156.43 ± 7.21 C: 155.65 ± 7.27	VR: 61.97 ± 8.67 C: 63.76 ± 6.76	Wii Fit	No intervention	2	6	TUG
Yves J. Gschwind (Australia)	63/61	VR: 82.5 ± 7 C: 80.2 ± 6.5	VR: 161.8 ± 7.7 C: 162.5 ± 10.1	VR: 69.8 ± 12.3 C: 70.1 ± 13.9	Wii Fit	No intervention	3	16	TUG; FTSST

^a^: The two C-Mill reports were based on independent participant samples and differed in sample size, intervention duration, and reported outcomes. No outcome data were included more than once in any meta-analysis. Note: NR indicates that the information was not reported in the original study.

**Table 4:** VR TUG Egger test results.

Std_Eff	Coefficient	Std. Err.	t	*p* > |t|	[95% Confidence Interval]
Slope	−0.2739071	0.2779566	−0.99	0.344	−0.8795225 0.3317084
bias	−1.05796	0.8648705	−1.22	0.245	−2.942351 0.8264313

## Data Availability

No new data were created or analyzed in this study.

## Supplementary Materials

The following supporting information can be downloaded at: https://www.mdpi.com/article/10.3390/healthcare14152409/s1. PRISMA 2020 Main Checklist [59]; S1. Summary of findings and certainty of evidence (GRADE); S2. Forest plot of sensitivity analysis for the effect of VR training on grip strength; S3. Forest plot of sensitivity analysis for the effect of VR training on five-times sit-to-stand test; S4. Forest plot of sensitivity analysis for the effect of VR training on gait speed; S5. Forest plot of subgroup analysis for the effect of WBV training on TUG; S6. Forest plot of subgroup analysis for the effect of WBV training on gait Speed; S7. Forest plot of sensitivity analysis for the effect of VR training on TUG.
