# Supplementary material for: Effects of Virtual Reality Training and Whole-Body Vibration Training on Fall-Related Functional Outcomes in Older Adults: A Systematic Review and Meta-Analysis
Source: Healthcare (Basel). 2026 Aug 5;14(15):2409. doi: 10.3390/healthcare14152409 (PMC13465516; doi:10.3390/healthcare14152409)

PRISMA 2020 Main Checklist

Topic	No.	Item	Locationwhereitemisreported
**TITLE**			
**Title**	1	Identify the report as a systematic review.	Lines 2–4
**ABSTRACT**			
**Abstract**	2	See the PRISMA 2020 for Abstracts checklist	Abstract, Lines 31–50; see PRISMA 2020 for Abstracts checklist below
**INTRODUCTION**			
**Rationale**	3	Describe the rationale for the review in the context of existing knowledge.	Lines 53–90
**Objectives**	4	Provide an explicit statement of the objective(s) or question(s) the review addresses.	Lines 91–97
**METHODS**			
**Eligibility criteria**	5	Specify the inclusion and exclusion criteria for the review and how studies were grouped for the syntheses.	Lines 116–132 and 186–187
**Information sources**	6	Specify all databases, registers, websites, organisations, reference lists and other sources searched or consulted to identify studies. Specify the date when each source was last searched or consulted.	Lines 105–112
**Search strategy**	7	Present the full search strategies for all databases, registers and websites, including any filters and limits used.	Table 1, Line 113
**Selection process**	8	Specify the methods used to decide whether a study met the inclusion criteria of the review, including how many reviewers screened each record and each report retrieved, whether they worked independently, and if applicable, details of automation tools used in the process.	Lines 134–137
**Data collection process**	9	Specify the methods used to collect data from reports, including how many reviewers collected data from each report, whether they worked independently, any processes for obtaining or confirming data from study	Lines 138–144

Topic	No.	Item	Locationwhereitemisreported
		investigators, and if applicable, details of automation tools used in the process.	
**Data items**	10a	List and define all outcomes for which data were sought. Specify whether all results that were compatible with each outcome domain in each study were sought (e.g. for all measures, time points, analyses), and if not, the methods used to decide which results to collect.	Lines 141–143 (selection among compatible measures/time points not specified)
	10b	List and define all other variables for which data were sought (e.g. participant and intervention characteristics, funding sources). Describe any assumptions made about any missing or unclear information.	Lines 138–140 (assumptions for missing/unclear information not reported)
**Study risk of bias assessment**	11	Specify the methods used to assess risk of bias in the included studies, including details of the tool(s) used, how many reviewers assessed each study and whether they worked independently, and if applicable, details of automation tools used in the process.	Lines 146–151
**Effect measures**	12	Specify for each outcome the effect measure(s) (e.g. risk ratio, mean difference) used in the synthesis or presentation of results.	Lines 175–182
**Synthesis methods**	13a	Describe the processes used to decide which studies were eligible for each synthesis (e.g. tabulating the study intervention characteristics and comparing against the planned groups for each synthesis (item 5)).	Lines 116–144 and 186–187
	13b	Describe any methods required to prepare the data for presentation or synthesis, such as handling of missing summary statistics, or data conversions.	Not reported
	13c	Describe any methods used to tabulate or visually display results of individual studies and syntheses.	Lines 173–174; results displayed in Figures 3–8
	13d	Describe any methods used to synthesize results and provide a rationale for the choice(s). If meta-analysis was performed, describe the model(s), method(s) to identify the presence and extent of statistical heterogeneity, and software package(s) used.	Lines 173–198

Topic	No.	Item	Locationwhereitemisreported
	13e	Describe any methods used to explore possible causes of heterogeneity among study results (e.g. subgroup analysis, meta-regression).	Lines 168–171 and 183–196
	13f	Describe any sensitivity analyses conducted to assess robustness of the synthesized results.	Lines 168–171
**Reporting bias assessment**	14	Describe any methods used to assess risk of bias due to missing results in a synthesis (arising from reporting biases).	Lines 158–160 and 173–174
**Certainty assessment**	15	Describe any methods used to assess certainty (or confidence) in the body of evidence for an outcome.	Lines 152–157
**RESULTS**			
**Study selection**	16a	Describe the results of the search and selection process, from the number of records identified in the search to the number of studies included in the review, ideally using a flow diagram.	Lines 201–208; Figure 2
	16b	Cite studies that might appear to meet the inclusion criteria, but which were excluded, and explain why they were excluded.	Not reported (no citations to apparently eligible excluded studies)
**Study characteristics**	17	Cite each included study and present its characteristics.	Lines 210–220; Tables 2 and 3
**Risk of bias in studies**	18	Present assessments of risk of bias for each included study.	Figure 1, Lines 161–166
**Results of individual studies**	19	For all outcomes, present, for each study: (a) summary statistics for each group (where appropriate) and (b) an effect estimate and its precision (e.g. confidence/credible interval), ideally using structured tables or plots.	Lines 239–399; Figures 3–7
**Results of syntheses**	20a	For each synthesis, briefly summarise the characteristics and risk of bias among contributing studies.	Lines 210–220 and 239–399; Figure 1, Lines 161–166 (partially reported)
	20b	Present results of all statistical syntheses conducted. If meta-analysis was done, present for each the summary estimate and its precision (e.g. confidence/credible interval) and measures of statistical heterogeneity. If comparing groups, describe the direction of the effect.	Lines 239–399; Figures 3–7

Topic	No.	Item	Locationwhereitemisreported
	20c	Present results of all investigations of possible causes of heterogeneity among study results.	Lines 260–273 and 376–388; Supplementary Figures S5–S6
	20d	Present results of all sensitivity analyses conducted to assess the robustness of the synthesized results.	Lines 221–236 and 304–306; Supplementary Figures S2–S4 and S7
**Reporting biases**	21	Present assessments of risk of bias due to missing results (arising from reporting biases) for each synthesis assessed.	Table 4 and Figure 8, Lines 401–404
**Certainty of evidence**	22	Present assessments of certainty (or confidence) in the body of evidence for each outcome assessed.	Supplementary Material S1
**DISCUSSION**			
**Discussion**	23a	Provide a general interpretation of the results in the context of other evidence.	Lines 406–503
	23b	Discuss any limitations of the evidence included in the review.	Lines 505–516
	23c	Discuss any limitations of the review processes used.	Not explicitly reported
	23d	Discuss implications of the results for practice, policy, and future research.	Lines 419–422 and 524–530
**OTHER INFORMATION**			
**Registration and protocol**	24a	Provide registration information for the review, including register name and registration number, or state that the review was not registered.	Lines 100–103
	24b	Indicate where the review protocol can be accessed, or state that a protocol was not prepared.	PROSPERO registration CRD420251250139, Lines 100–103
	24c	Describe and explain any amendments to information provided at registration or in the protocol.	Not reported
**Support**	25	Describe sources of financial or non-financial support for the review, and the role of the funders or sponsors in the review.	Lines 539–542 (funding sources reported; funder role not specified)
**Competing interests**	26	Declare any competing interests of review authors.	Lines 531–532

Topic	No.	Item	Locationwhereitemisreported
**Availability of data, code and other materials**	27	Report which of the following are publicly available and where they can be found: template data collection forms; data extracted from included studies; data used for all analyses; analytic code; any other materials used in the review.	Lines 543–546

PRIMSA Abstract Checklist

Topic	No.	Item	Reported?
**TITLE**			
**Title**	1	Identify the report as a systematic review.	Yes
**BACKGROUND**			
**Objectives**	2	Provide an explicit statement of the main objective(s) or question(s) the review addresses.	Yes
**METHODS**			
**Eligibility criteria**	3	Specify the inclusion and exclusion criteria for the review.	Yes
**Information sources**	4	Specify the information sources (e.g. databases, registers) used to identify studies and the date when each was last searched.	Yes
**Risk of bias**	5	Specify the methods used to assess risk of bias in the included studies.	Yes
**Synthesis of results**	6	Specify the methods used to present and synthesize results.	Yes
**RESULTS**			
**Included studies**	7	Give the total number of included studies and participants and summarise relevant characteristics of studies.	Yes
**Synthesis of results**	8	Present results for main outcomes, preferably indicating the number of included studies and participants for each. If meta-analysis was done, report the summary estimate and confidence/credible interval. If comparing groups, indicate the direction of the effect (i.e. which group is favoured).	Yes
**DISCUSSION**			
**Limitations of evidence**	9	Provide a brief summary of the limitations of the evidence included in the review (e.g. study risk of bias, inconsistency and imprecision).	Yes
**Interpretation**	10	Provide a general interpretation of the results and important implications.	Yes
**OTHER**			
**Funding**	11	Specify the primary source of funding for the review.	Yes
**Registration**	12	Provide the register name and registration number.	Yes

From: Page MJ, McKenzie JE, Bossuyt PM, Boutron I, Hoffmann TC, Mulrow CD, et al. The PRISMA 2020 statement: an updated guideline for reporting systematic reviews. MetaArXiv.

2020, September 14. DOI: 10.31222/osf.io/v7gm2. For more information, visit: www.prisma-statement.org

Supplementary S1. Summary of findings and certainty of evidence (GRADE)

Outcome	No.ofstudies(participants)	Effectestimate(MD,95%CI)	Certaintyofevidence(GRADE)
a VR Timed Up and Go Test（TUG）	14 RCTs (n = 560)	−1.13 (−1.70 to −0.55)	Moderate ⊕ ⊕ ⊕◯
b WBV Timed Up and Go Test（TUG）	8 RCTs (n = 392)	−3.92 (−6.36 to −1.48)	Moderate ⊕ ⊕ ⊕◯
c VR Tinetti score	3 RCTs (n = 100)	0.84 (0.46 to 1.21)	Moderate ⊕ ⊕ ⊕◯
d WBV Tinetti score	5 RCTs (n = 278)	0.53 (−0.21 to 1.27)	Moderate ⊕ ⊕ ⊕◯
e VR Handgrip Strength	3 RCTs (n = 209)	3.49 (0.46 to 6.52)	Low ⊕ ⊕◯◯
f WBV Handgrip Strength	2 RCTs (n = 48)	−0.77 (−6.17 to 4.62)	Low ⊕ ⊕◯◯
g VR Five Times Sit-to-Stand Test	3 RCTs (n = 164)	−3.95 (−9.00 to 1.10)	Very Low ⊕◯◯◯
h WBV Five Times Sit-to-Stand Test	4 RCTs (n = 193)	−1.20 (−2.03 to −0.38)	Moderate ⊕ ⊕ ⊕◯
i VR Gait Speed	3 RCTs (n = 197)	0.16 (0.07 to 0.24)	Low ⊕ ⊕◯◯
j WBV Gait Speed	5 RCTs (n = 255)	0.04 (−0.10 to 0.19)	Very Low ⊕◯◯◯

a:Downgraded one level for inconsistency because substantial heterogeneity was observed in the primary analysis (I^2=69%). Although heterogeneity decreased to 16% after exclusion of Marek Zak et al., the pooled effect remained similar in magnitude, direction, and statistical significance, indicating that the overall finding was robust but that between-study variability remained a concern.

b:Downgraded one level for inconsistency because substantial between-study heterogeneity was observed (I^2=70%). The subgroup analysis by intervention duration also showed differing effects between shorter- and longer-duration interventions, suggesting that intervention duration may partly explain the variability across studies.

c:Downgraded one level for risk of bias because some contributing trials raised concerns in one or more RoB 2 domains. No downgrading was applied for inconsistency or imprecision because no statistical heterogeneity was observed (I^2=0%) and the confidence interval was relatively narrow and excluded the null effect.

d:Downgraded one level for imprecision because the confidence interval crossed the line of no effect and included both little or no effect and a potentially meaningful improvement in Tinetti score.

e:Downgraded one level for risk of bias because some of the contributing trials raised concerns in one or more RoB 2 domains, and one level for inconsistency because substantial heterogeneity was present (I^2=73%). After exclusion of Park et al., heterogeneity decreased to 0%, while the effect estimate was attenuated and the confidence interval approached the null, indicating sensitivity to the inclusion of this study.

f:Downgraded two levels for very serious imprecision because only two small trials with 48 participants were included, and the wide confidence interval crossed the line of no effect and encompassed both potentially important benefit and harm.
g:Downgraded one level for risk of bias because some contributing trials raised concerns in one or more RoB 2 domains, one level for inconsistency because substantial heterogeneity was observed (I^2=67%), and one level for imprecision because the confidence interval crossed the line of no effect and included both a potentially important benefit and little or no effect. The sensitivity analysis also yielded only borderline statistical significance after exclusion of Campo-Prieto et al.

h:Downgraded one level for risk of bias because some contributing trials were judged to have some concerns or a high risk of bias in one or more RoB 2 domains. No additional downgrading was applied for inconsistency or imprecision because heterogeneity was low to moderate (I^2=39%) and the confidence interval did not cross the line of no effect.

i:Downgraded one level for risk of bias because some contributing trials raised concerns in one or more RoB 2 domains, and one level for inconsistency because substantial heterogeneity was observed (I^2=69%). Heterogeneity decreased to 0% after exclusion of Sadeghi et al., indicating that the pooled estimate was influenced by an individual study.

j:Downgraded one level for risk of bias because some contributing trials raised concerns in one or more RoB 2 domains, one level for inconsistency because considerable heterogeneity was observed (I^2=91%) and the direction of effect differed between age subgroups, and one level for imprecision because the confidence interval crossed the line of no effect and included both possible improvement and possible worsening of gait speed.

Supplementary S2 Forest plot of sensitivity analysis for the effect of VR training on grip strength.

Supplementary Figure S2. Forest plot of VR training on grip strength. VR training significantly improved grip strength compared with control intervention (MD = 1.76, 95% CI: 0.03 to 3.49, P = 0.05), with no observed heterogeneity (I^2 = 0%).

	VR			Control				Mean Difference
Study or Subgroup	Mean	SD	Total	Mean	SD	Total	Weight	IV, Fixed, 95% CI
Pablo Campo-Prieto2022	20.5	6.18	13	16.95	5.97	11	12.7%	3.55 [-1.32, 8.42]
Steven Phu2019	21.5	5	63	20	5	50	87.3%	1.50 [-0.36, 3.36]
**Total (95% CI)**			**76**			**61**	**100.0%**	**1.76 [0.03, 3.49]**

Heterogeneity: Chi² = 0.59, df = 1 (P = 0.44); I² = 0%
Test for overall effect: Z = 1.99 (P = 0.05)

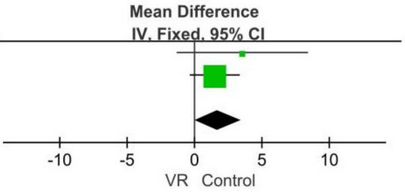

Supplementary S3 Forest plot of sensitivity analysis for the effect of VR training on five-times sit-to-stand test.

Supplementary Figure S3. Forest plot of the effect of virtual reality (VR) training on the five-times sit-to-stand test. After sensitivity analysis, VR training showed a borderline significant reduction in completion time compared with the control intervention (MD = −1.29, 95% CI: −2.58 to 0.00, P = 0.05), with no observed heterogeneity among the included studies (I^2 = 0%).

	VR			Control				Mean Difference
Study or Subgroup	Mean	SD	Total	Mean	SD	Total	Weight	IV, Fixed, 95% CI
Lisa Sheehy l2025	9.3	1.55	8	12.4	8.4	8	4.7%	-3.10 [-9.02, 2.82]
Yves J. Gschwind2015	12.1	3.7	63	13.3	3.8	61	95.3%	-1.20 [-2.52, 0.12]
**Total (95% CI)**			**71**			**69**	**100.0%**	**-1.29 [-2.58, -0.00]**

Heterogeneity: Chi² = 0.38, df = 1 (P = 0.54); I² = 0%
Test for overall effect: Z = 1.96 (P = 0.05)

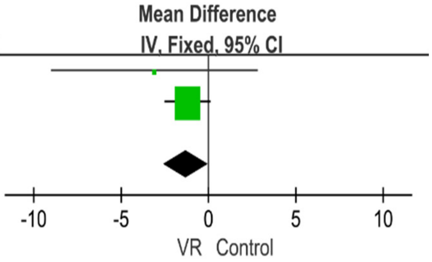

Supplementary S4 Forest plot of sensitivity analysis for the effect of VR training on gait speed.

Supplementary Figure S4. Forest plot of the effect of virtual reality (VR) training on gait speed. After sensitivity analysis, VR training significantly improved gait speed compared with the control intervention (MD = 0.11, 95% CI: 0.06 to 0.17, P < 0.0001), with no observed heterogeneity among the included studies (I^2 = 0%).

	VR			Control				Mean Difference
Study or Subgroup	Mean	SD	Total	Mean	SD	Total	Weight	IV, Fixed, 95% CI
Ehab M Abd El-Kafy2024	1.26	0.13	27	1.16	0.14	27	52.3%	0.10 [0.03, 0.17]
Steven Phu2019	0.94	0.18	63	0.81	0.22	50	47.7%	0.13 [0.05, 0.21]
**Total (95% CI)**			**90**			**77**	**100.0%**	**0.11 [0.06, 0.17]**

Heterogeneity: Chi² = 0.32, df = 1 (P = 0.57); I² = 0%
Test for overall effect: Z = 4.30 (P < 0.0001)

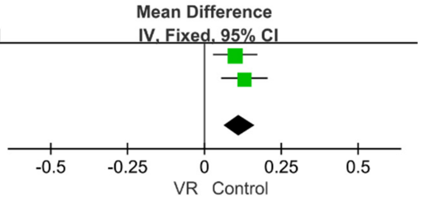

Supplementary S5 Forest plot of subgroup analysis for the effect of WBV training on TUG.

Supplementary Figure S5. Forest plot of subgroup analysis according to intervention duration for the effect of whole-body vibration (WBV) training on the timed up-and-go test. In studies with an intervention duration of ≤8 weeks, WBV training significantly reduced TUG completion time compared with the control intervention (MD = −4.93, 95% CI: −7.29 to −2.57, P < 0.0001), with moderate heterogeneity (I^2 = 45%). In studies with an intervention duration >8 weeks, the reduction was not statistically significant (MD = −1.60, 95% CI: −3.58 to 0.32, P = 0.10), with no observed heterogeneity (I^2 = 0%).

	Experimental			Control				Mean Difference
Study or Subgroup	Mean	SD	Total	Mean	SD	Total	Weight	IV, Random, 95% CI
**2.2.1 WBV>8 week**								
1Charlotte Beaudart2013	17.86	4	31	19.51	3.74	31	22.8%	-1.65 [-3.58, 0.28]
1Harri Sievänen2014	34.7	34.7	8	27.3	11.5	7	0.9%	7.40 [-18.11, 32.91]
**Subtotal (95% CI)**			**39**			**38**	**23.6%**	**-1.60 [-3.52, 0.32]**
Heterogeneity: Tau² = 0.00; Chi² = 0.48, df = 1 (P = 0.49); I² = 0%								
Test for overall effect: Z = 1.63 (P = 0.10)								
**2.2.2 WBV≤8 week**								
1Francisco Ålvarez-Barbosa2014	9.7	3.4	11	15.7	6.08	11	15.1%	-6.00 [-10.12, -1.88]
1Ivan Bautmans2005	12	3.7	10	14.3	7.1	11	13.2%	-2.30 [-7.08, 2.48]
1Li Zhang2013	21.34	4.42	19	30.39	9.42	18	13.1%	-9.05 [-13.83, -4.27]
1Mercé Sitja-Rabert 2014	21.14	29.14	59	18.52	11.66	63	6.9%	2.62 [-5.35, 10.59]
1Olivier Bruyere2005	36.4	16.3	16	33.9	29.9	20	2.3%	2.50 [-12.85, 17.85]
1Ross D Pollock2012	18.83	1.91	38	24.47	1.92	39	25.7%	-5.64 [-6.50, -4.78]
**Subtotal (95% CI)**			**153**			**162**	**76.4%**	**-4.93 [-7.29, -2.57]**
Heterogeneity: Tau² = 3.37; Chi² = 9.01, df = 5 (P = 0.11); I² = 45%								
Test for overall effect: Z = 4.10 (P < 0.0001)								
**Total (95% CI)**			**192**			**200**	**100.0%**	**-3.92 [-6.36, -1.48]**

Heterogeneity: Tau² = 5.85; Chi² = 23.28, df = 7 (P = 0.002); I² = 70%
Test for overall effect: Z = 3.15 (P = 0.002)
Test for subgroup differences: Chi² = 4.61, df = 1 (P = 0.03), I² = 78.3%

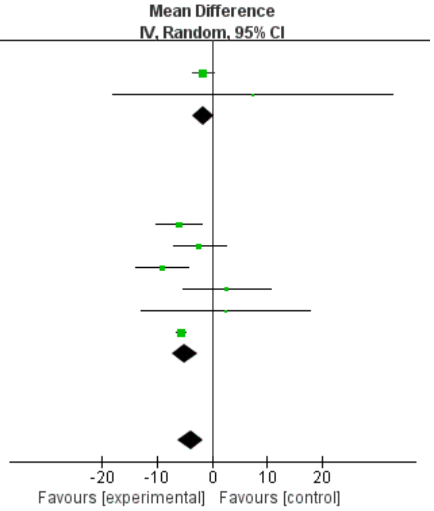

Supplementary S6 Forest plot of subgroup analysis for the effect of WBV training on gait Speed.

Supplementary Figure S6. Forest plot of subgroup analysis according to mean participant age for the effect of whole-body vibration (WBV) training on gait speed. In studies with a mean participant age ≥70 years, WBV training significantly improved gait speed compared with the control intervention (MD = 0.14, 95% CI: 0.05 to 0.23, P = 0.004), with moderate heterogeneity (I^2 = 57%). In studies with a mean participant age <70 years, the effect did not reach statistical significance (MD = −0.07, 95% CI: −0.14 to 0.00, P = 0.06), with no observed heterogeneity (I^2 = 0%).

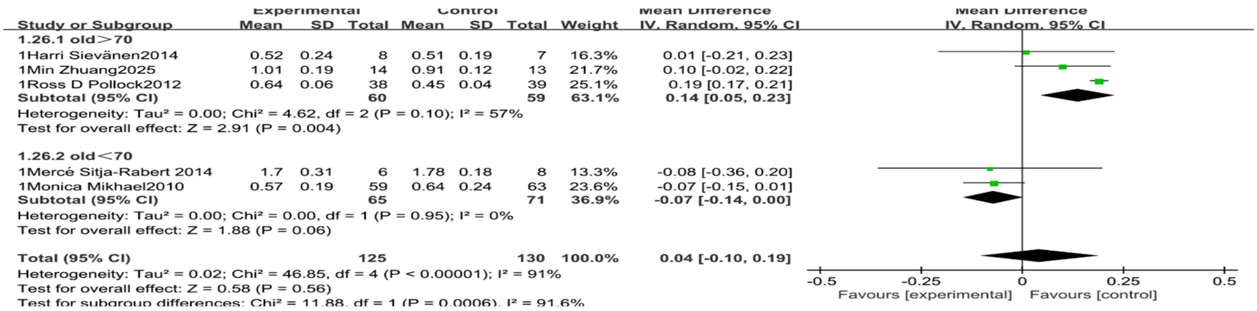

Supplementary S7 Forest plot of sensitivity analysis for the effect of VR training on TUG.

Supplementary Figure S7. Forest plot of the sensitivity analysis for the effect of VR training on TUG after exclusion of Marek Zak et al. VR training remained associated with a significant reduction in TUG completion time compared with the control intervention (MD = −1.12, 95% CI: −1.38 to −0.85, P<0.00001), while heterogeneity decreased from 69% to 16%.

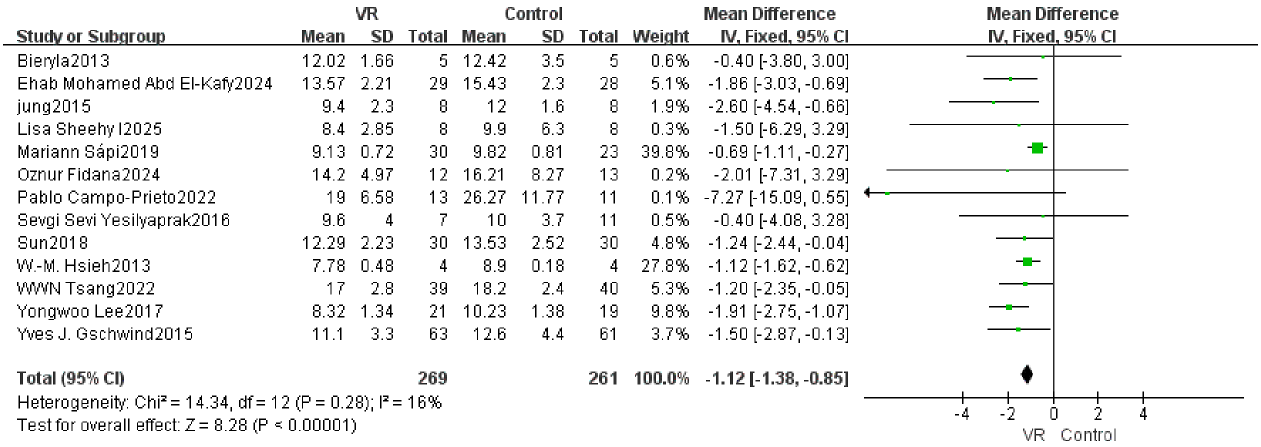

Supplement: Supplementary file 1 [file healthcare-14-02409-s001.zip › healthcare-4431341-supplementary.pdf]
